# PZLAST: an ultra-fast amino acid sequence similarity search server against public metagenomes

**DOI:** 10.1093/bioinformatics/btab492

**Published:** 2021-07-08

**Authors:** Hiroshi Mori, Hitoshi Ishikawa, Koichi Higashi, Yoshiaki Kato, Toshikazu Ebisuzaki, Ken Kurokawa

**Affiliations:** Department of Informatics, National Institute of Genetics, Mishima, Shizuoka 411-8540, Japan; PEZY Computing, K. K., 5F Chiyoda Ogawamachi Crosta, Chiyoda-ku, Tokyo 101-0052, Japan; Department of Informatics, National Institute of Genetics, Mishima, Shizuoka 411-8540, Japan; Computational Astrophysics Laboratory, RIKEN, Wako, Saitama, Japan; Computational Astrophysics Laboratory, RIKEN, Wako, Saitama, Japan; Department of Informatics, National Institute of Genetics, Mishima, Shizuoka 411-8540, Japan

## Abstract

**Summary:**

: Similarity searches of amino acid sequences against the public metagenomic data can provide users insights about the function of sequences based on the environmental distribution of similar sequences. However, a considerable reduction in the amount of data or the accuracy of the result was necessary to conduct sequence similarity searches against public metagenomic data, because of the vast data size more than Terabytes. Here, we present an ultra-fast service for the highly accurate amino acid sequence similarity search, called PZLAST, which can search the user’s amino acid sequences to several Terabytes of public metagenomic sequences in ∼10–20 min. PZLAST accomplishes its search speed by using PEZY-SC2, which is a Multiple Instruction Multiple Data many-core processor. Results of PZLAST are summarized by the ontology-based environmental distribution of similar sequences. PZLAST can be used to predict the function of sequences and mine for homologs of functionally important gene sequences.

**Availability and implementation:**

PZLAST is freely accessible at https://pzlast.riken.jp/meta without requiring registration.

**Supplementary information:**

Supplementary data are available at *Bioinformatics* online.

## 1 Introduction

Shotgun metagenomic sequence data are rapidly accumulating in public sequence databases (Kyrpides *et al.*, 2016). Sequence similarity searches against metagenomic sequence data provide important information about environmental distributions of similar sequences, which can be used for the finding of functionally important gene homologs and for the functional inference of sequences. Several groups provide web-based sequence similarity search services that can be used without downloading the huge (more than Terabytes) reference data (Levi *et al.*, 2018; Mitchell *et al.*, 2020). In these web applications, reference sequence data are still large, and this in turn requires reducing the reference data (Levi *et al.*, 2018) or compressing the data by sequence assembling to reduce the calculation time (Mitchell *et al.*, 2020). However, down-sampling of sequences wipes out minor sequences in the sample, and assembling sequences eliminates abundance information of the sequence in the sample. To elucidate the environmental distribution of similar sequences, e.g. these reduced reference data would make results need to be treated carefully because the results could contain artificial biases for gene distributions.

This study presents a web-based service called PZLAST that provides extremely fast and highly accurate amino acid sequence similarity searches against several Terabytes of public metagenomic amino acid sequence data.

## 2 Materials and methods

### 2.1 PZLAST algorithm

PZLAST uses multiple PEZY-SC2s, which are Multiple Instruction Multiple Data (MIMD) many-core processors (Hishinuma and Nakata, 2019). In a Single Instruction Multiple Data processor, all threads execute the same instructions for different data. On the other hand, in an MIMD processor, each thread executes different instructions for different data. Such MIMD feature of PEZY-SC2 is suitable to realize various calculation stages in PZLAST. A rapid sequence similarity search is achieved distributing its task to multiple PEZY-SC2s in a data parallel way, and utilizing a large number of (15 872) threads in a PEZY-SC2. The basis of the sequence similarity search algorithm of PZLAST is similar to the CLAST algorithm (Yano *et al.*, 2014). CLAST is designed for conducting BLAST-like (Camacho *et al.*, 2009) DNA sequence similarity searches with graphic processing units. In PZLAST, the CLAST algorithm was modified mainly in the following two aspects: (i) use amino acid sequences instead of DNA sequences to improve the sensitivity and (ii) swap the query and reference sequences to improve the search speed. The details of the PZLAST algorithm are described in Supplementary Data.

### 2.2 Reference sequence data

The reference amino acid sequence data for PZLAST were obtained from the MicrobeDB.jp database (https://microbedb.jp). The MicrobeDB.jp pipeline utilizes Prodigal with a metagenome option (Hyatt *et al.*, 2012) for predicting protein-coding genes from public metagenomic sequence read data from the DNA DataBank of Japan Read Archive (Ogasawara *et al.*, 2020). Because predicting genes from the short read metagenomic sequences are sometimes difficult, some genes in short reads are possibly unpredicted in this method. Although the number of hits in PZLAST results could be slightly underrepresented, PZLAST uses the predicted amino acid sequences as a reference for similarity searches. The size of reference amino acid sequence data is about 2.5 Terabytes (∼1.7 trillion amino acids in 42.3 billion sequences), which was obtained from 4339 shotgun metagenome samples. In MicrobeDB.jp, each metagenome sample is automatically annotated with the sampled environment information by using Metagenome and Microbes Environmental Ontology (MEO) (https://bioportal.bioontology.org/ontologies/MEO). All the amino acid sequence data and metadata of the PZLAST reference database can be obtained from http://palaeo.nig.ac.jp/Resources/PZLAST/AASequences/.

### 2.3 Tool comparison

To evaluate the validity of PZLAST search results, we compared the precision, recall rates, maximal RAM (Random Access Memory) consumption and calculation time of sequence similarity searches between PZLAST and two other tools (DIAMOND and MMseqs2) (Buchfink *et al.*, 2015; Steinegger and Söding, 2017), which are commonly used in metagenomic analyses. However, generally speaking, these tools are difficult to search against several Terabytes sequence database because of the huge memory requirements (Ye *et al.*, 2019). Therefore, we used only an initial 1 000 000, 10 000 000, 100 000 000 and 1 000 000 000 sequences of the reference database (the data sizes were 63 Megabytes, 630 Megabytes, 6.3 Gigabytes and 59 Gigabytes, respectively) for the tool comparison. A gyrase B sequence of *Enterococcus* (648 residues, WP_002288364) and a type I polyketide synthase sequence of *Streptomyces* (7746 residues, WP_010981856) were used for queries of the tool comparison, as a broadly conserved protein across many taxa and a large protein that is conserved only in specific taxa, respectively. Also, the highly conserved 15 protein sequences of *Escherichia coli* K-12 MG1655 with various lengths were used for the comparison of the precision and recall rates. The precision and recall rates of the 1 000 000 sequences data were calculated based on the SSEARCH result as ground truth data because SSEARCH performs a rigorous Smith–Waterman alignment (Pearson, 1991). Precision and recall rates were only calculated using the 1 000 000 sequences data because of the difficulty to conduct SSEARCH against huge reference sequence data. The version and search parameters of each tool were described in Supplementary Table S1. We used a Dell PowerEdge R840 PC server (four Xeon Gold 6242 2.8 GHz 16 cores CPUs, 768 Gigabytes RAM) running 64 threads for the performance comparison of SSEARCH, DIAMOND and MMseqs2.

## 3 Results

### 3.1 Tool comparison result

The precision and recall rates of PZLAST and the other two tools are described in Table 1. The relationships of the precision and recall rates of 17 proteins in the range of *E*-value threshold 1e−10 to 1 are indicated in Supplementary Figure S2. The statistics of PZLAST are as good as or even better than those of the other two tools in the *E*-value 1e−8 (Table 1), indicating that PZLAST can provide reliable search results in the cases of moderately or highly similar sequences. The maximal RAM consumption and calculation time of these three tools are described in Supplementary Tables S2 and S3, respectively. These two tables indicate that for both RAM consumption and calculation time, PZLAST is relatively unsusceptible against reference data increase. Thus, PZLAST can provide users with fast and accurate sequence similarity searches against the huge size of metagenomic sequence data.

**Table 1.** Precision and recall rates comparison among three tools

	Gyrase B (303 hits in SSEARCH)		Polyketide synthase (61 hits in SSEARCH)	
Tool name	Precision % (hits)	Recall % (hits)	Precision % (hits)	Recall % (hits)
DIAMOND	100 (215 hits)	70.95 (215 hits)	100 (54 hits)	88.52 (54 hits)
MMseqs2	100 (224 hits)	73.92 (224 hits)	96.55 (58 hits)	91.8 (58 hits)
PZLAST	100 (221 hits)	72.93 (221 hits)	91.04 (67 hits)	100 (67 hits)

### 3.2 PZLAST web service

**Fig. 1.**
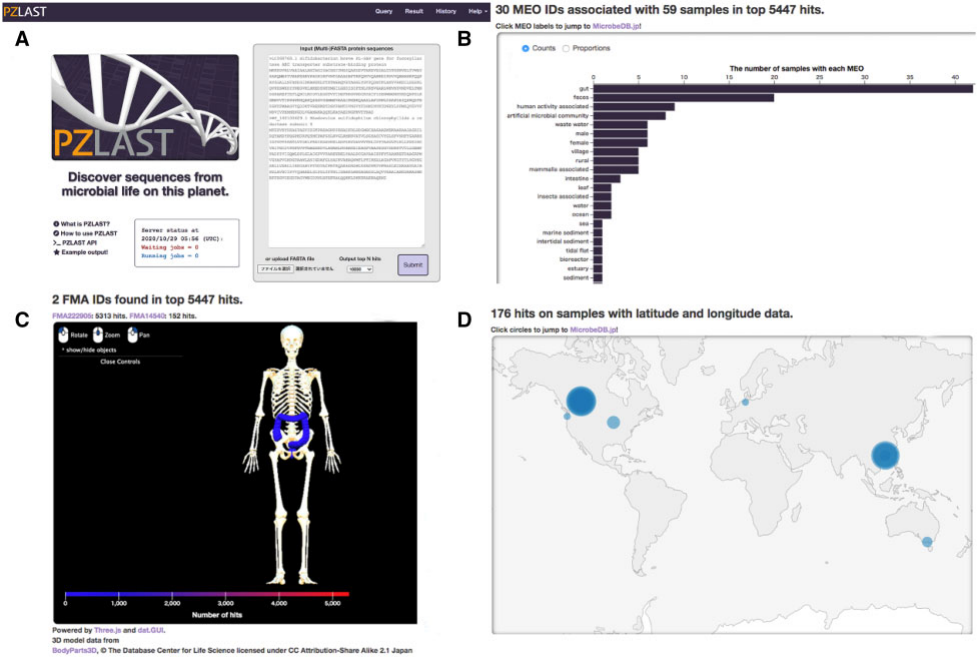
Overview of the PZLAST web service. (**A**) PZLAST web interface. (**B**) Summary of a PZLAST result based on MEO classes. (**C**) Summary of a PZLAST result based on FMA classes using a BodyParts3D model. In this figure, only the colon is highlighted, which indicates that the homologs of the query sequence were found in the colon. (**D**) Summary of a PZLAST result based on geographical distribution. Blue circles indicate the locations of the homologs

A user can submit a maximum of 10 000 sequences per submission (Fig. 1A). Although PZLAST supports multiple job scheduling, currently the number of simultaneous jobs was limited to one due to the total amount of our available hardware resources. Just after a user submits a query, one unique URL for the search result is shown. The search takes ∼10–20 min per query, and then the server displays its results on the unique URL. The search results are the top 10 000 hits per query in the default and summarized based on MEO classes (Fig. 1B), Foundational Model of Anatomy ontology (FMA) classes (Fig. 1C) and geographical distribution (Fig. 1D). The MEO summarization shows the environmental distribution of similar genes, and the FMA summarization indicates the distribution of similar genes in the human body (Fujieda and Okubo, 2016). For example, in Figure 1, the query sequence was that of fucosyllactose ABC transporter substrate-binding protein from Bifidobacterium which is important for the adaptation of bifidobacteria in the infant gut (Matsuki *et al.*, 2016). According to Figure 1B and C, this protein was mainly found in gut, confirming the PZLAST search validity. At the moment of the original study, no information regarding how common is this gene worldwide, since the original study was a comparative genome analysis among many isolated bifidobacteria from Japanese infants. The PZLAST result in Figure 1D indicates that this gene can be found in human gut microbiome samples from other countries. As stated above, the web browser-based PZLAST search gives us visually fruitful results. In addition to it, PZLAST also provides a REST API service to search sequences and obtain its results, which will be useful for users who need the search result without manual operations.

In this comparison, we used the 1 000 000 metagenomic sequences as the reference database. We calculated the precision and recall rates of three tools in the case of *E*-value threshold 1e−8. The SSEARCH result of the *E*-value 1e−8 was ground truth data for this comparison.

## 4 Conclusion

PZLAST provides users with ultra-fast and highly accurate sequence similarity searches against public metagenomic amino acid sequences without any reference data reduction. PZLAST can be used for functional prediction of sequences, and for homolog mining of functionally important gene sequences.

## Supplementary Material

btab492_Supplementary_Data
