# Supplementary material for: PZLAST: an ultra-fast amino acid sequence similarity search server against public metagenomes
Source: Bioinformatics. 2021 Jul 8;37(21):3944–6. doi: 10.1093/bioinformatics/btab492 (PMC8570820; doi:10.1093/bioinformatics/btab492)
Supplement: btab492_Supplementary_Data [file btab492_supplementary_data.docx]

**PZLAST: an ultra-fast amino acid sequence similarity search server against public metagenomes**

Hiroshi Mori^1#*^, Hitoshi Ishikawa^2#^, Koichi Higashi^1#^, Yoshiaki Kato^3^, Toshikazu Ebisuzaki^3^, Ken Kurokawa^1^

Included in this supplementary data:

1. Supplementary Figure 1: PZLAST sequence search workflow
2. Supplementary Figure 2: Precision-Recall plots of three tools
3. Supplementary Table 1: Tool version and parameters for the tool comparison
4. Supplementary Table 2: Maximum RAM consumption comparison among three tools
5. Supplementary Table 3: Wall-clock calculation time (sec) comparison among three tools
6. Supplementary Notes
7. PZLAST sequence search algorithm
8. Comparison of the precision and recall rates of 17 proteins
9. Cautious points when searching sequences using PZLAST


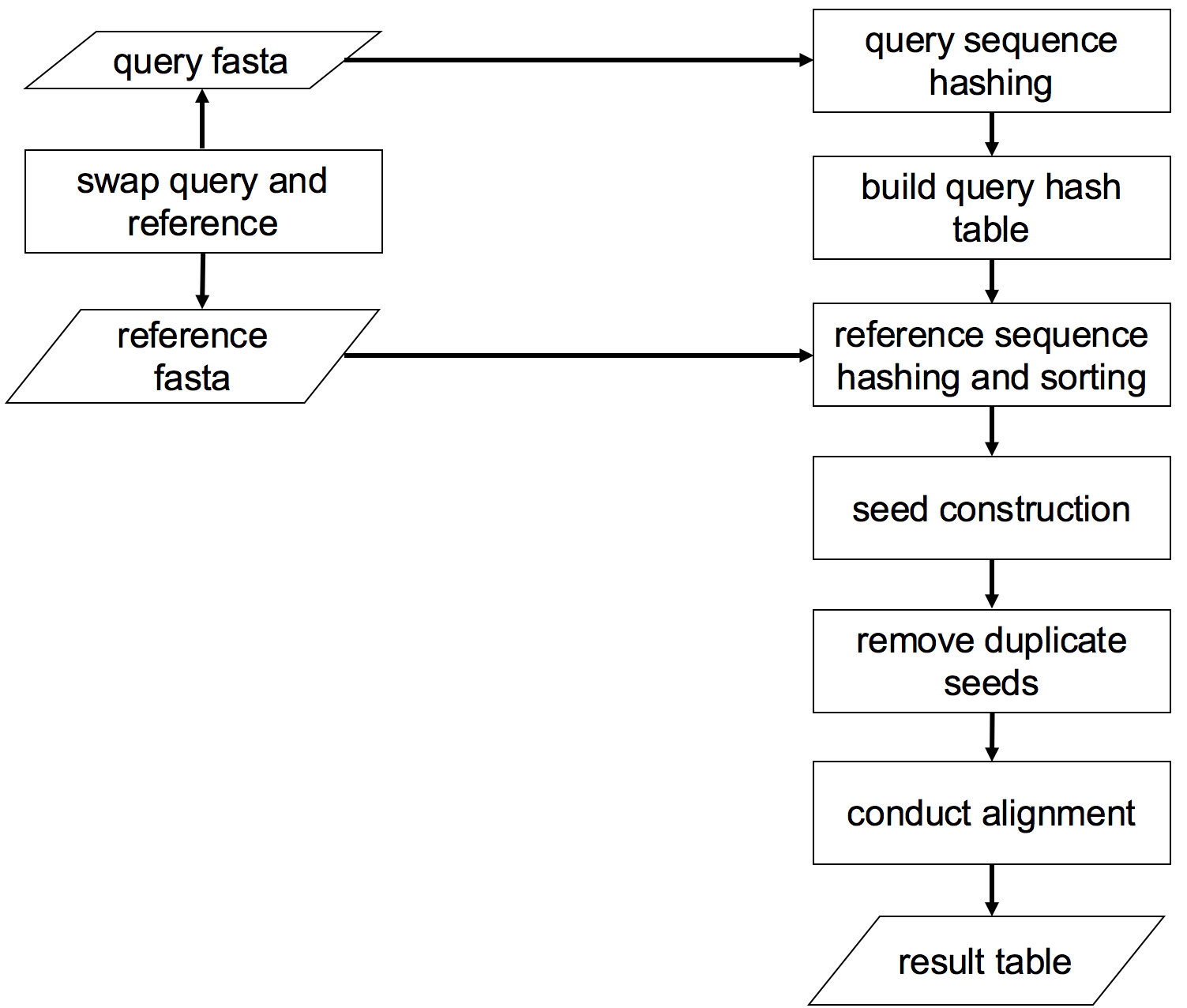


**Supplementary Figure 1: PZLAST sequence search workflow.**

The query fasta indicates the user-submitted amino acid sequence data. The reference fasta indicates the public metagenomic amino acid sequence data. The detail of the PZLAST sequence search algorithm is described in Supplementary Notes.

**
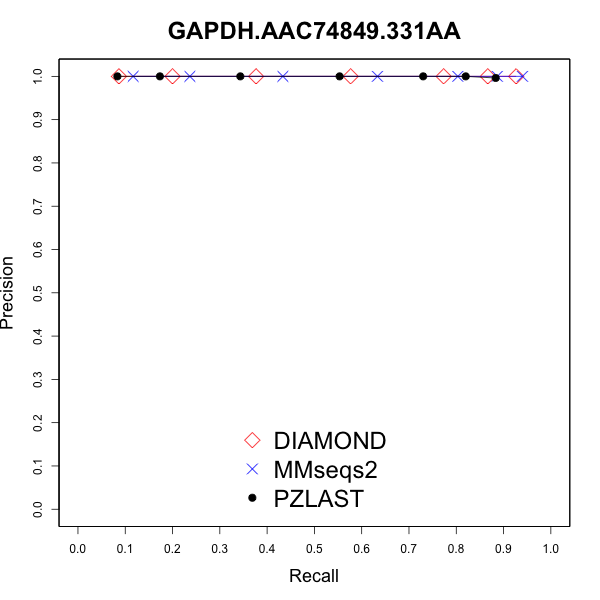
**
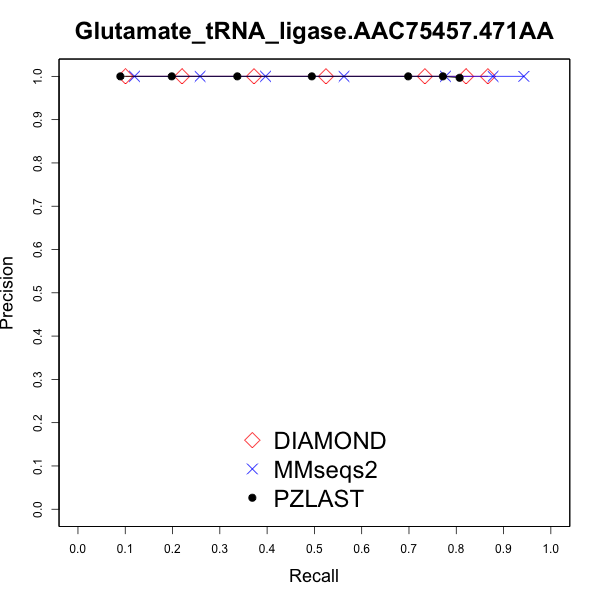
**
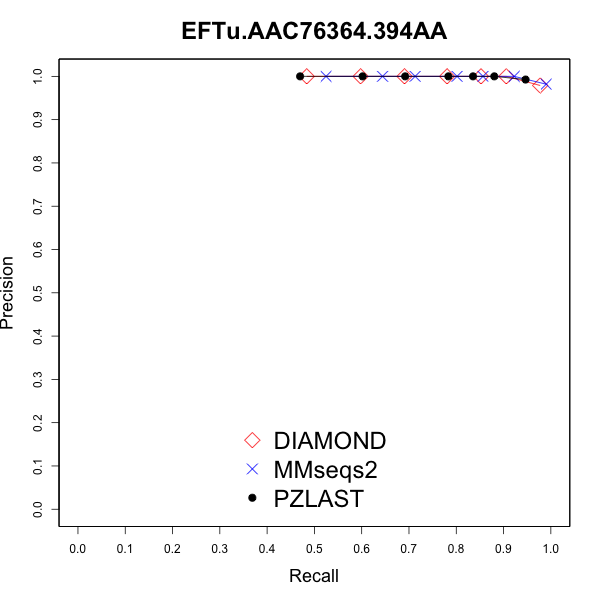
**A B C


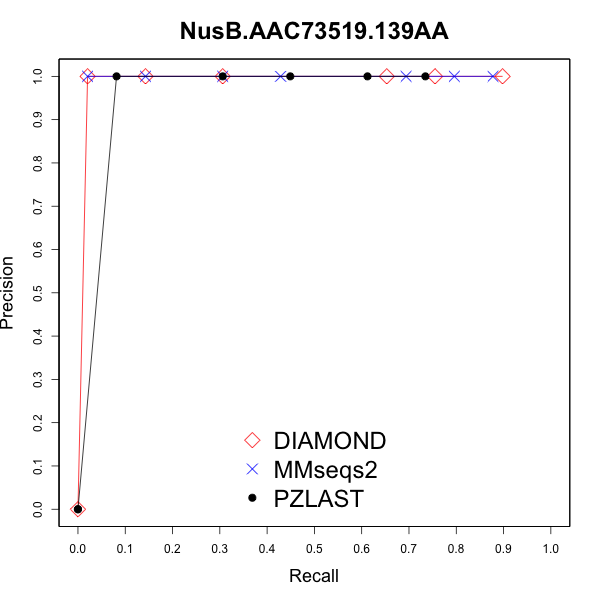

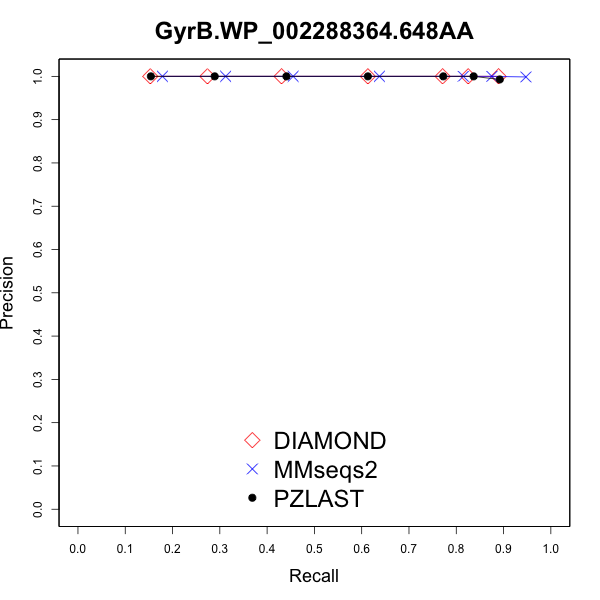

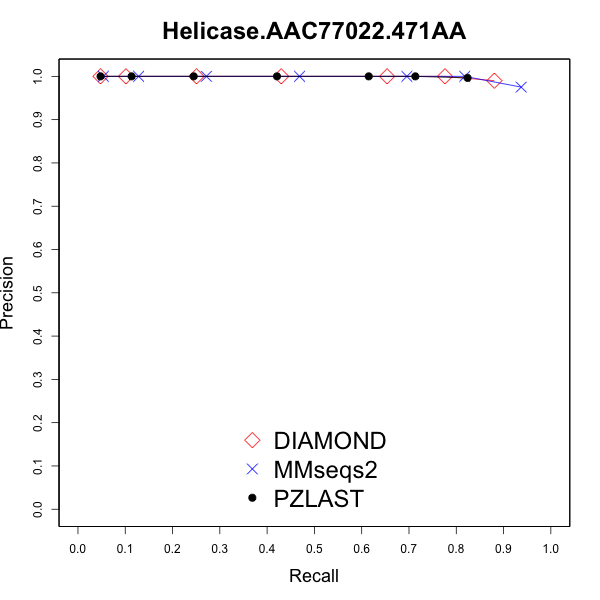
D E F


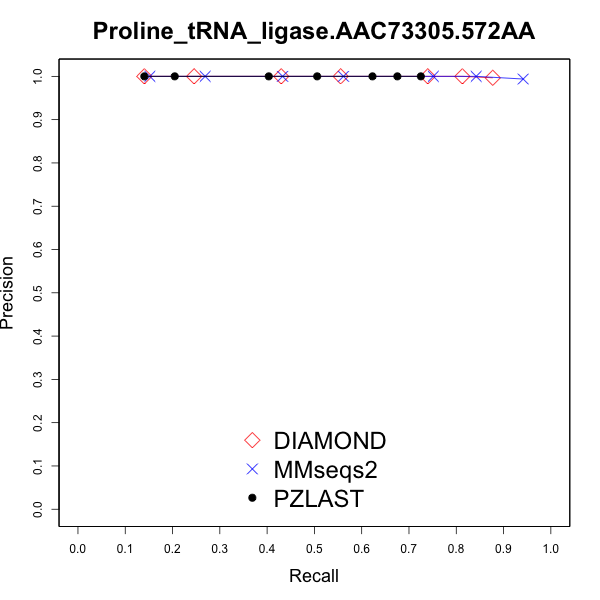

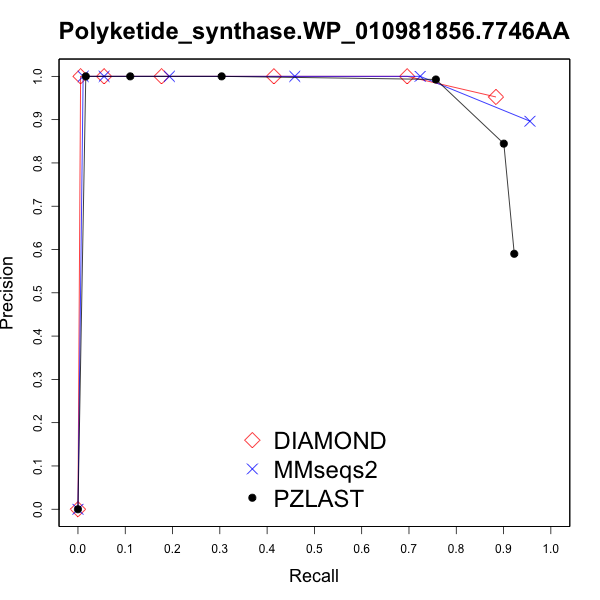

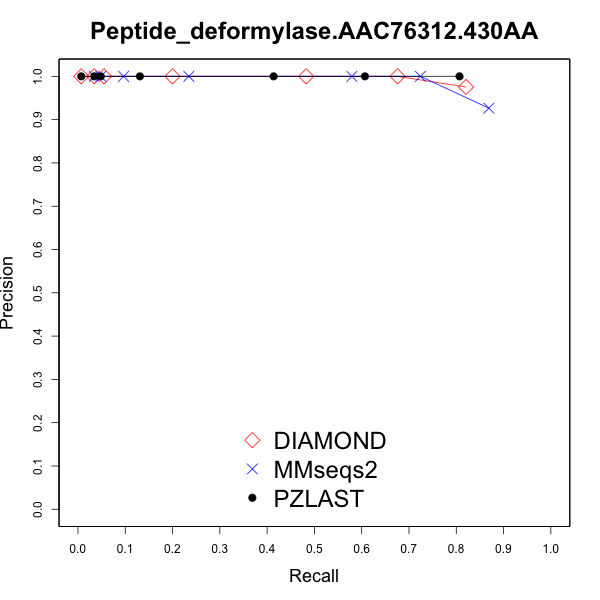


G H I

**Supplementary Figure 2: Precision-Recall plots of three tools.**

The relationships between the precision rate and the recall rate of the three tools against one million reference metagenomic sequences are plotted in the seven E-value thresholds (1e-10, 1e-8, 1e-6, 1e-4, 1e-2, 1e-1, and 1).


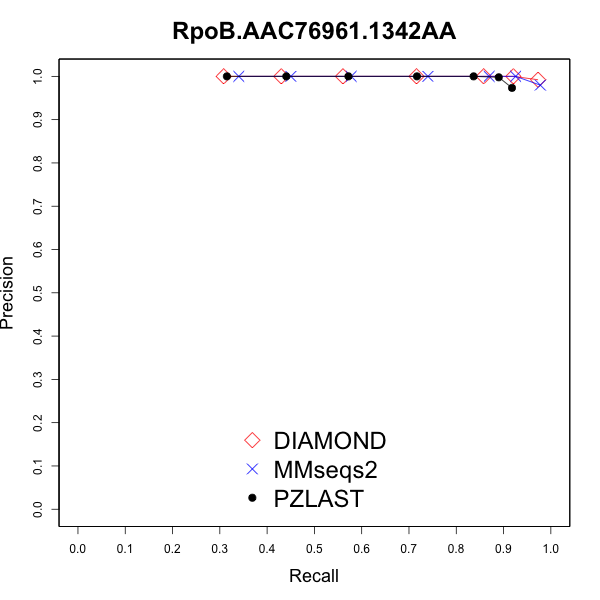

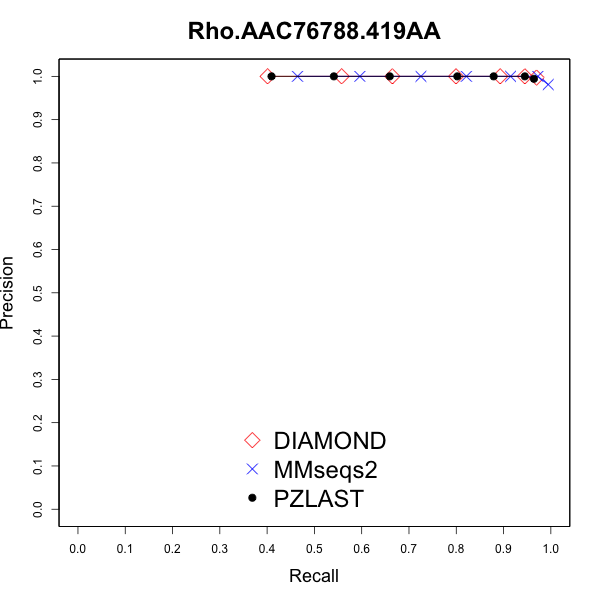

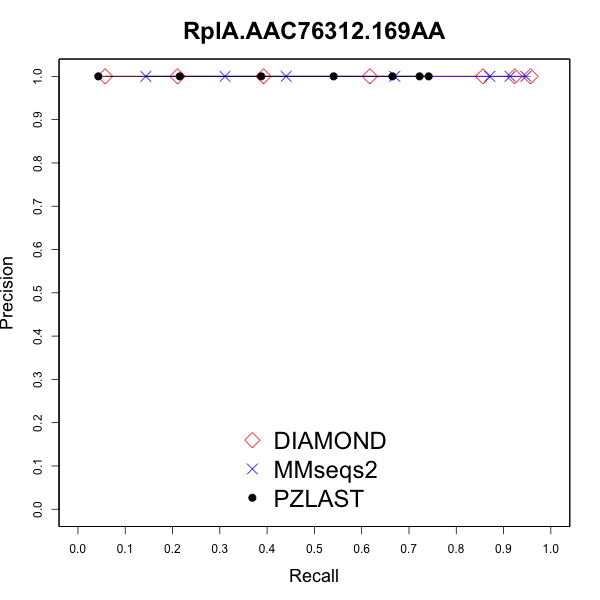
J K L


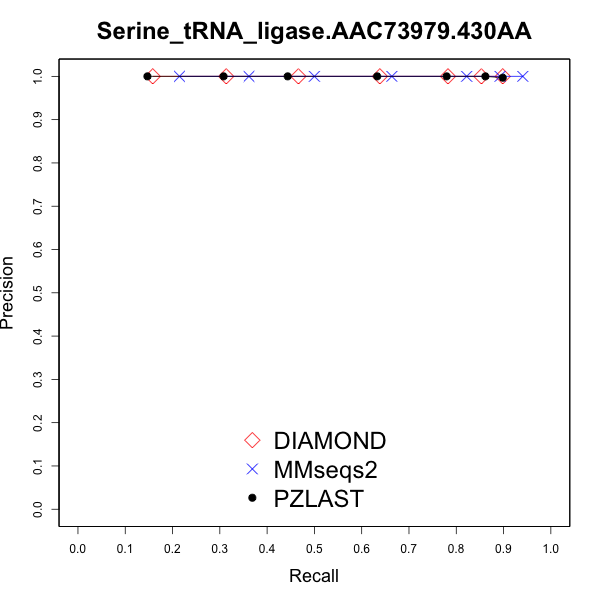

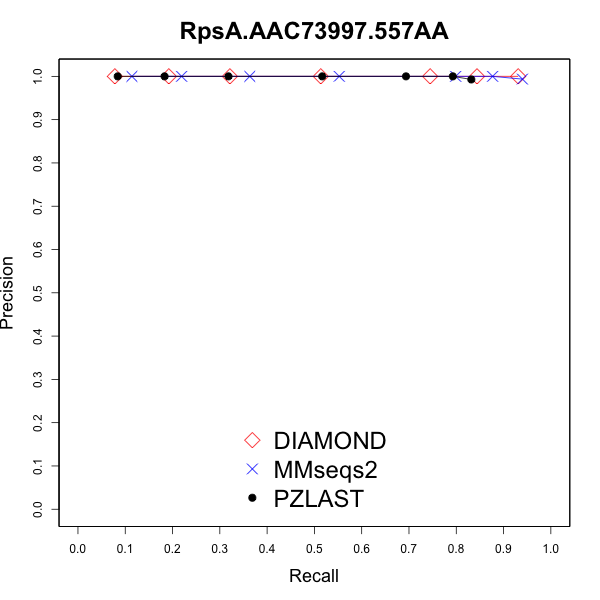

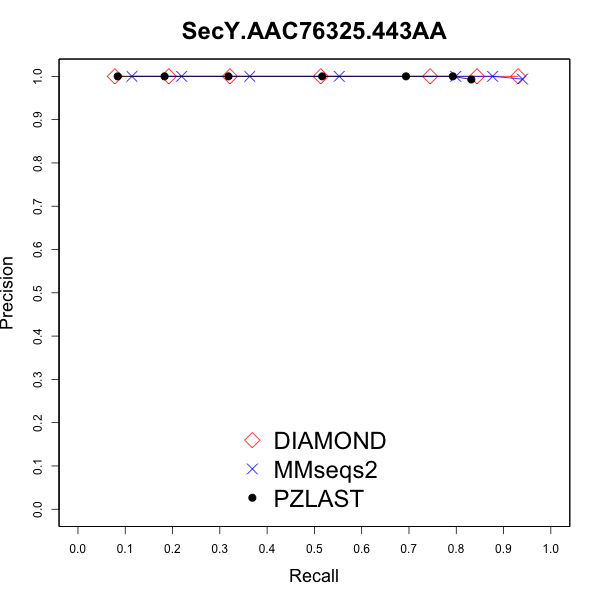
M N O


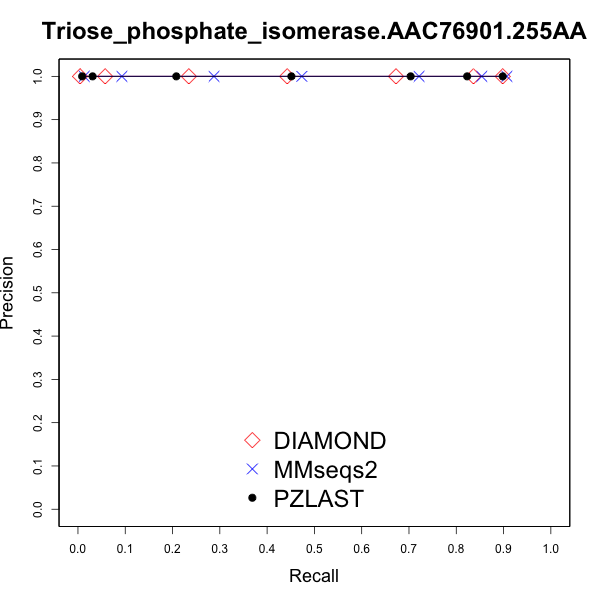

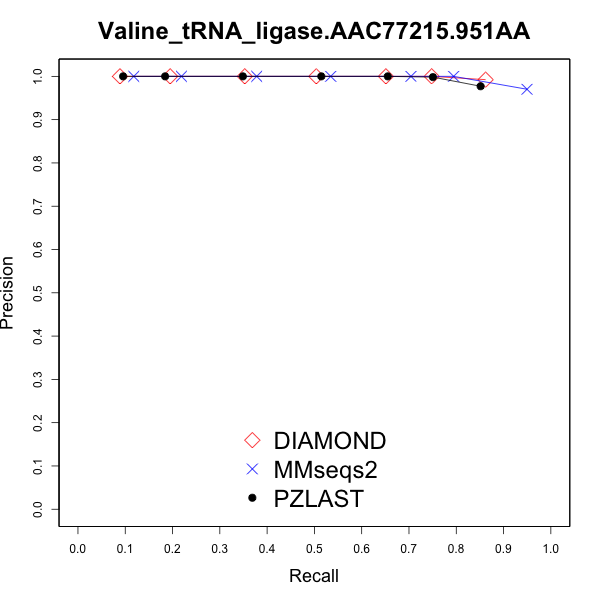
P Q

**Supplementary Figure 2: Precision-Recall plots of three tools (continued from the previous page).**

| Tool name | Version | Parameters |
| --- | --- | --- |
| SSEARCH | 36.3.8g | -s BL62 -E 1e-8 |
| DIAMOND | 0.9.31 | blastp --sensitive --evalue 1e-8 --max-target-seqs 10000 --outfmt 6 |
| MMseqs2 | 11 | search -s 7.5 --max-seqs 10000 -e 1e-8 |

**Supplementary Table 1: Tool version and parameters for the tool comparison.**

The performance comparison of SSEARCH, DIAMOND, and MMseqs2 was conducted on a Dell PowerEdge R840 PC server running 64 threads (four Xeon Gold 6242 2.8GHz 16 cores CPUs, 768 Gigabytes RAM).

|  |  | reference size | | | |
| --- | --- | --- | --- | --- | --- |
| tool name | query protein | 1M | 10M | 100M | 1,000M |
| DIAMOND | gyrase B | 250.16 | 1,657.10 | 7,716.91 | 18,797.08 |
| MMseqs2 | gyrase B | 0.9 | 3,699.34 | 41,502.28 | 244,012.52 |
| PZLAST | gyrase B | < 8,000 | < 8,000 | < 8,000 | < 8,000 |
| DIAMOND | polyketide synthase | 211.93 | 3,041.83 | 11,886.89 | 18,685.63 |
| MMseqs2 | polyketide synthase | 0.9 | 3,699.31 | 41,502.60 | 243,912.02 |
| PZLAST | polyketide synthase | < 8,000 | < 8,000 | < 8,000 | < 8,000 |

**Supplementary Table 2: Maximum RAM consumption comparison among three tools.**

Maximum RAM consumptions (Megabytes) were compared using two protein sequences against 1M (million), 10M, 100M, and 1000M reference metagenomic sequences among three tools. As for PZLAST, since almost all calculation tasks are off-loaded onto PEZY-SC2s, 8 Gigabytes CPU RAM is sufficient regardless the size of reference.

|  |  | reference size | | | |
| --- | --- | --- | --- | --- | --- |
| tool name | query protein | 1M | 10M | 100M | 1,000M |
| DIAMOND | gyrase B | 1.23 | 11.1 | 89.13 | 891.64 |
| MMseqs2 | gyrase B | 7.87 | 47.39 | 532.31 | 5,820.21 |
| PZLAST | gyrase B | 70.38 | 70.3 | 60.35 | 90.86 |
| DIAMOND | polyketide synthase | 4.51 | 10.46 | 106.01 | 1,060.10 |
| MMseqs2 | polyketide synthase | 9.05 | 54.77 | 568.16 | 6,300.24 |
| PZLAST | polyketide synthase | 90.51 | 70.38 | 70.86 | 111.06 |

**Supplementary Table 3: Wall-clock calculation time (sec) comparison among three tools.**

Calculation time were compared using two protein sequences against 1M (million), 10M, 100M, and 1000M reference metagenomic sequences among three tools.

**Supplementary Notes**

**a. PZLAST sequence search algorithm**

PZLAST conducts amino acid vs. amino acid sequence similarity searches. In the sequence alignment construction, PZLAST uses BLOSUM62 as a substitution matrix. The PZLAST workflow is described in Supplementary Figure 1. Initially, because the reference metagenomic sequences are much larger (2.5 Terabytes) than the user-submitted query sequences (less than 1 Megabytes), PZLAST swaps the query and reference sequences. When the size of the input data is small, this method of swapping reference and query sequences is effective in improvement of the search speed, which is mainly due to the reduced time necessary to build the hash table, sort seeds, and construct alignment. Since the number of hashes created from the PZLAST reference sequences is much larger than that from the query sequences, the time for sorting the hash values to create a hash table is significantly large when the hash table is made of the reference. This sorting time was efficiently reduced by swapping the query and reference sequences, and building the hash table from the query sequences. The default hash length of PZLAST is 4-mer, which provides sensitive sequence similarity searches. Because the length of amino acid sequences in our read-based CDSs database is generally short, to improve the sensitivity of sequence search, PZLAST skips the eliminate-isolate-seeds-step in the CLAST algorithm, and extends alignment if one or more seeds are found between the reference and query sequences. PZLAST conducts local alignment between the query and reference sequences. The default E-value threshold of PZLAST is 1e-8. Other parts of the PZLAST algorithm are based on CLAST which is designed almost similar to BLAST [Yano et al. 2014]. Thus PZLAST has inherited the ability to provide highly accurate sequence similarity searches from both CLAST and BLAST.

**b. Comparison of the precision and recall rates of 17 proteins.**

As described in the Results section of our manuscript, the precision and recall rates of PZLAST are as good as or even better than those of the other two tools in the realistic condition (E-value threshold 1e-8). We further investigated the relationships between the precision and recall rate of the three tools in the seven different E-value thresholds (1e-10, 1e-8, 1e-6, 1e-4, 1e-2, 1e-1, and 1) against one million reference metagenomic sequences by plotting these values (Supplementary Figure 2). The SSEARCH results with an E-value threshold of 0.1 were used as ground truth data, and the following 17 proteins were selected to calculate the precision and recall rates. GyrB and type I polyketide synthase that were used in Table 1, and 15 highly conserved proteins from *E. coli* K-12 MG1655: GTP-binding elongation factor protein (an abbreviated protein name is EF-Tu, an accession number is AAC76364, a protein length is 394 residues), Glyceraldehyde-3-phosphate dehydrogenase (GAPDH, AAC74849, 331 residues), Glutamate tRNA ligase (AAC75457, 471 residues), DNA helicase (AAC77022, 471 residues), Transcription antitermination protein (NusB, AAC73519, 139 residues), Peptide deformylase (AAC76312, 169 residues), Proline tRNA ligase (AAC73305, 572 residues), Transcription termination factor (Rho, AAC76788, 419 residues), 50S ribosomal subunit protein L1 (RplA, AAC76312, 169 residues), DNA-directed RNA polymerase beta subunit (RpoB, AAC76961, 1342 residues), 30S ribosomal subunit protein S1 (RpsA, AAC73997, 557 residues), Membrane protein translocase (SecY, AAC76325, 443 residues), Serine tRNA ligase (AAC73979, 430 residues), Triose-phosphate isomerase (AAC76901, 255 residues), and Valine tRNA ligase (AAC77215, 951 residues). The precision and recall rates of the two proteins (AAC73519 and WP_010981856) are 0 in the case of the 1e-10 E-value threshold because we could not find any hit better than the threshold (the exception is MMseqs2 in AAC73519). In most cases, PZLAST exhibits nearly the same result compared with the other two tools. However, the recall rates of PZLAST in some proteins (e.g., AAC73305, AAC73519, and AAC76312) do not exhibit the high values compared with the other two tools in cases of the high E-value thresholds. These small values of the recall rates of PZLAST in the high E-value thresholds are because of the l_mer setting (l_mer = 4) of PZLAST. In the seed search step of the PZLAST search, PZLAST finds a complete match of four residues between the query and a reference sequence. However, when the query and the reference sequence are far from, sometimes no complete match of four residues exists between them. Therefore, PZLAST cannot find some hits that SSEARCH founds in the case of the high E-value thresholds and the recall rates of PZLAST are not exhibit high values. This phenomenon is observed in the situation that PZLAST searches weakly similar sequences (about the E-value threshold > 1e-4). Because we set the E-value threshold as 1e-8 in the PZLAST web service, the search result of the PZLAST web service is not affected by this phenomenon.

**c. Cautious points when searching sequences using PZLAST**

When searching sequences using PZLAST, users should be careful of some query dependent strange search results. One example is, querying sequences containing low complexity regions (e.g., single amino acid repeat) can cause enormous amounts of hits. The reason for a large number of hits is that such low complex sequences are often artificially generated during the DNA sequencing reactions. The other example is, large amino acid sequences (e.g., polyketide synthase) usually are not found a hit with high query coverage. This false negative is because the reference amino acid sequences in PZLAST were predicted from short reads, and sometimes not have full protein length. Therefore, the query coverage-based filtering is generally not useful for filtering the PZLAST hit results. To avoid this false negative, users need to split such a long query sequence to short fragments. More optimal solution for this problem will be considered in the near future.

The environment distribution of metagenomic samples in the current PZLAST reference database is enriched to the human gut and seawater metagenomes, and not so many samples are included from the hot spring, wastewater, or other environments. This biased distribution of sampling environment is mainly due to the research effort bias of metagenome researchers. But this bias is expected to ease by adding more samples to the reference sequence database in future updates.
